# Proteome-scale understanding of relationship between homo-repeat enrichments and protein aggregation properties

**DOI:** 10.1371/journal.pone.0206941

**Published:** 2018-11-06

**Authors:** Oxana V. Galzitskaya, Miсhail Yu. Lobanov

**Affiliations:** Group of Bioinformatics, Institute of Protein Research, Russian Academy of Science, Pushchino, Moscow Region, Russia; Russian Academy of Medical Sciences, RUSSIAN FEDERATION

## Abstract

Expansion of homo-repeats is a molecular basis for human neurological diseases. We are the first who studied the influence of homo-repeats with lengths larger than four amino acid residues on the aggregation properties of 1449683 proteins across 122 eukaryotic and bacterial proteomes. Only 15% of proteins (215481) include homo-repeats of such length. We demonstrated that RNA-binding proteins with a prion-like domain are enriched with homo-repeats in comparison with other non-redundant protein sequences and those in the PDB. We performed a bioinformatics analysis for these proteins and found that proteins with homo-repeats are on average two times longer than those in the whole database. Moreover, we are first to discover that as a rule, homo-repeats appear in proteins not alone but in pairs: hydrophobic and aromatic homo-repeats appear with similar ones, while homo-repeats with small, polar and charged amino acids appear together with different preferences. We elaborated a new complementary approach to demonstrate the influence of homo-repeats on their host protein aggregation properties. We have shown that addition of artificial homo-repeats to natural and random proteins results in intensification of aggregation properties of the proteins. The maximal effect is observed for the insertion of artificial homo-repeats with 5–6 residues, which is consistent with the minimal length of an amyloidogenic region. We have also demonstrated that the ability of proteins with homo-repeats to aggregate cannot be explained only by the presence of long homo-repeats in them. There should be other characteristics of proteins intensifying the aggregation property including such as the appearance of homo-repeats in pairs in the same protein. We are the first who elaborated a new approach to study the influence of homo-repeats present in proteins on their aggregation properties and performed an appropriate analysis of the large number of proteomes and proteins.

## Introduction

Eukaryotic and bacterial proteomes contain proteins bearing simple amino acid motifs including homo-repeats consisting of a single multiply repeated amino acid. The understanding of the amino acid tandem repeat function in different proteomes is one of the important tasks of molecular biology. It turned out that some homo-repeats play more important roles in the biological processes [1] and are associated with human diseases than it was previously recognized. Strong selection of homo-repeats in evolution for all proteomes has been demonstrated [2].

The question about the influence of homo-repeats in proteins on the increasing or decreasing the fraction of disordered residues was considered in several publications [3–7]. It was shown that the occurrence of homo-repeats with hydrophobic amino acids results in a decreasing fraction of disordered residues, at the same time this value for charge, polar and small amino acid residues increases. The maximum fraction of disordered residues was obtained for proteins with lysine and arginine homo-repeats, and the minimum value corresponds to valine and leucine homo-repeats [7]. The recent review by Darling and Uversky concentrates on the intrinsic disorder in proteins with pathogenic repeat expansions, considering only alanine and glutamine homo-repeats [8].

As we demonstrated earlier, that the minimal size of homo-repeats varies with amino acid types and proteomes. We have found that homo-repeats containing polar or small amino acids S, P, H, E, D, K, Q, and N are enriched in structural disorder as well as protein- and RNA-interactions. We observed that E, S, Q, G, L, P, D, A, and H homo-repeats are strongly associated with the occurrence in human diseases. Moreover, S, E, P, A, Q, D, and T homo-repeats are significantly enriched in neuronal proteins associated with autism and other disorders [2].

It was shown that proteins containing alanine repeats of ten and more residues were able to aggregate [9]. It should be stressed that expansion of homo-repeats is a molecular basis for at least 18 human neurological diseases. Several proteins were found to be associated with poly-A (alanine) developmental diseases (9 inherited human diseases) [8,10]: cleidocranial dysplasia (CCD, gene RUNX2), congenital central hypo-ventilation syndrome (CCHS, gene PHOX2B), hand–foot–genital syndrome (HFGS, gene HOXA13), blepharophimosis (BPEIS, gene FOXL2), oculopharyngeal muscular dystrophy (OPMD, gene PABPN1), infantile spasm syndrome (XLMR, gene ARX), X-linked mental retardation and abnormal genitalia (XLAG, gene ARX), X-linked mental retardation and growth hormone deficit (XLMR + GHD, gene SOX3), and holoprosencephaly (HPE, gene ZIC2) [10]. Expansion of poly-Q is implicated in several neurodegenerative diseases, including Huntington’s disease and several spinocerebellar ataxias. It should be noted that the length of the poly-Q repeat is critical to pathogenesis. Although a repeat of 40 glutamine residues is present in the forkhead box P2 transcription factor normal allele, the protein has not been found to be associated with a poly-Q disease [11].

Recently it has been found that local compositional enrichment within protein sequences affects the translation efficiency, abundance, half-life, subcellular localization, and molecular functions of proteins [12]. It should be mentioned several papers about aggregation propensity of the human [13], yeast [14] proteomes, and cytosolic *E*. *coli* proteome [15], but without consideration of homo-repeats.

One can suggest that the occurrence of homo-repeats in the protein sequence results in the increasing aggregation ability of the proteins. They are more aggregation-prone. It is well known that an increase in the number of PrP repeats induces spontaneous prion disease [16], whereas repeat deletion retards the disease and diminishes PrPSc formation [17]. *In vitro*, two extra copies of R2 repeat cause the N-terminal and Middle domains (NM) of SUP35 to aggregate with an abbreviated lag phase, whereas deletion of R2–R5 repeats extends the lag phase [18,19]. Therefore, a large number of repeats will facilitate the correct alignment of intermolecular contacts between protein molecules that drive amyloid formation [20].

Rapidly formed fibrils stimulate aggregation acting as seeds and can greatly decrease with increasing differences in the primary structure. A good example is immunoglobulin domains with different primary structures. It was shown that co-aggregation between different types of domains is not observed when the identity of the primary structure is below 30–40% [21]. The bioinformatics analysis of the tandem homologous domains in large multi-domain proteins revealed homology less than 40%, which probably indicates that the primary structure of proteins is arranged so as to avoid aggregation. One can conclude that modulation of the aggregation propensity is a driving force in protein evolution.

In this respect important questions arise: what lengths and type of homo-repeats can affect aggregation properties of their host proteins? What differences exist between the proteins with homo-repeats and without them? We are the first who have made a bioinformatics analysis of the influence of homo-repeats of different lengths on aggregation properties of their host proteins for the analysis covered all 20 amino acid residues and 122 proteomes.

## Results and discussion

### Systematic analysis of occurrence of homo-repeats in 1449683 proteins from 122 proteomes and in the different sets of proteins

To investigate the influence of homo-repeats on the aggregation properties of proteins we should define what length of homo-repeat is not random. In our previous analysis we demonstrated what length of amino acid residues is not random [2]. For each of 20 amino acids, this length was determined considering that the occurrence of such lengths of homo-repeats differs at least 10-fold between natural and expected occurrence in 122 proteomes. Therefore, for our analysis we considered the effect of only homo-repeats with the length larger than four amino acid residues (single-amino-acid tandem repeats) in the proteins on the aggregation properties of host proteins from 122 eukaryotic and bacterial proteomes. It should be noted that the lengths of five and six residues are the minimal lengths which are responsible for aggregation or can be considered as amyloidogenic regions [22,23] although dipeptide IlePhe can form amyloid fibrils [24].

In some proteomes there are not sufficient proteins containing homo-repeats for statistics (see Table 1, [25]), therefore we combined all proteins for analysis, and the database includes 1 449 683 (Np) proteins.

**Table 1:** Number of proteins having at least one pair of homo-repeats.

	C	M	F	I	L	V	W	Y	A	G	T	S	Q	N	E	D	H	R	K	P
**C**	*7*	1	3	2	*25*	4	0	3	22	*49*	10	20	11	8	11	8	8	6	7	20
**M**	1	*8*	3	1	*25*	2	0	0	19	7	13	22	27	19	*30*	16	5	6	13	11
**F**	3	3	**79**	*17*	*76*	19	0	8	52	56	45	78	51	72	38	23	12	23	*107*	38
**I**	2	1	*17*	**52**	*56*	*22*	0	**31**	13	25	42	47	30	*92*	16	16	16	6	25	10
**L**	*25*	*25*	*76*	*56*	*372*	44	1	*33*	*1014*	351	261	579	265	190	*540*	180	56	184	158	425
**V**	4	2	19	*22*	44	*67*	2	2	147	117	55	108	53	46	61	56	11	37	27	46
**W**	0	0	0	0	1	2	*1*	0	5	5	3	5	1	1	3	3	0	0	0	1
**Y**	3	0	8	**31**	*33*	2	0	**25**	11	8	30	23	18	*64*	14	19	4	0	*29*	11
**A**	22	19	52	13	*1014*	147	5	11	**5230**	**4957**	*1579*	**3843**	**4016**	1017	*2024*	*1548*	**975**	*893*	548	**3178**
**G**	*49*	7	56	25	351	117	5	8	**4957**	**5339**	*1468*	**3349**	**3217**	*1327*	*1674*	*1385*	**868**	*792*	417	**2528**
**T**	10	13	45	42	261	55	3	30	*1579*	*1468*	**3313**	**3313**	**3117**	**3236**	*1114*	*1096*	*529*	209	355	*1267*
**S**	20	22	78	47	579	108	5	23	**3843**	**3349**	**3313**	**5735**	**4801**	**3614**	*2316*	*1833*	**1166**	*733*	*990*	*2922*
**Q**	11	27	51	30	265	53	1	18	**4016**	**3217**	**3117**	**4801**	**8080**	**4202**	*1698*	*1524*	**1523**	361	509	**3157**
**N**	8	19	72	*92*	190	46	1	*64*	1017	*1327*	**3236**	**3614**	**4202**	**6486**	*1212*	*1435*	*667*	117	*1256*	854
**E**	11	*30*	38	16	*540*	61	3	14	*2024*	*1674*	*1114*	*2316*	*1698*	*1212*	**3427**	**2196**	*312*	*472*	*1180*	*1565*
**D**	8	16	23	16	180	56	3	19	*1548*	*1385*	*1096*	*1833*	*1524*	*1435*	**2196**	**1714**	*302*	*343*	*804*	*1001*
**H**	8	5	12	16	56	11	0	4	**975**	**868**	*529*	**1166**	**1523**	*667*	*312*	*302*	**617**	79	92	*675*
**R**	6	6	23	6	184	37	0	0	*893*	*792*	209	*733*	361	117	*472*	*343*	79	**443**	*234*	*549*
**K**	7	13	*107*	25	158	27	0	*29*	548	417	355	*990*	509	*1256*	*1180*	*804*	92	*234*	**1793**	422
**P**	20	11	38	10	425	46	1	11	**3178**	**2528**	*1267*	*2922*	**3157**	854	*1565*	*1001*	*675*	*549*	422	**4692**

**Fig 1:**
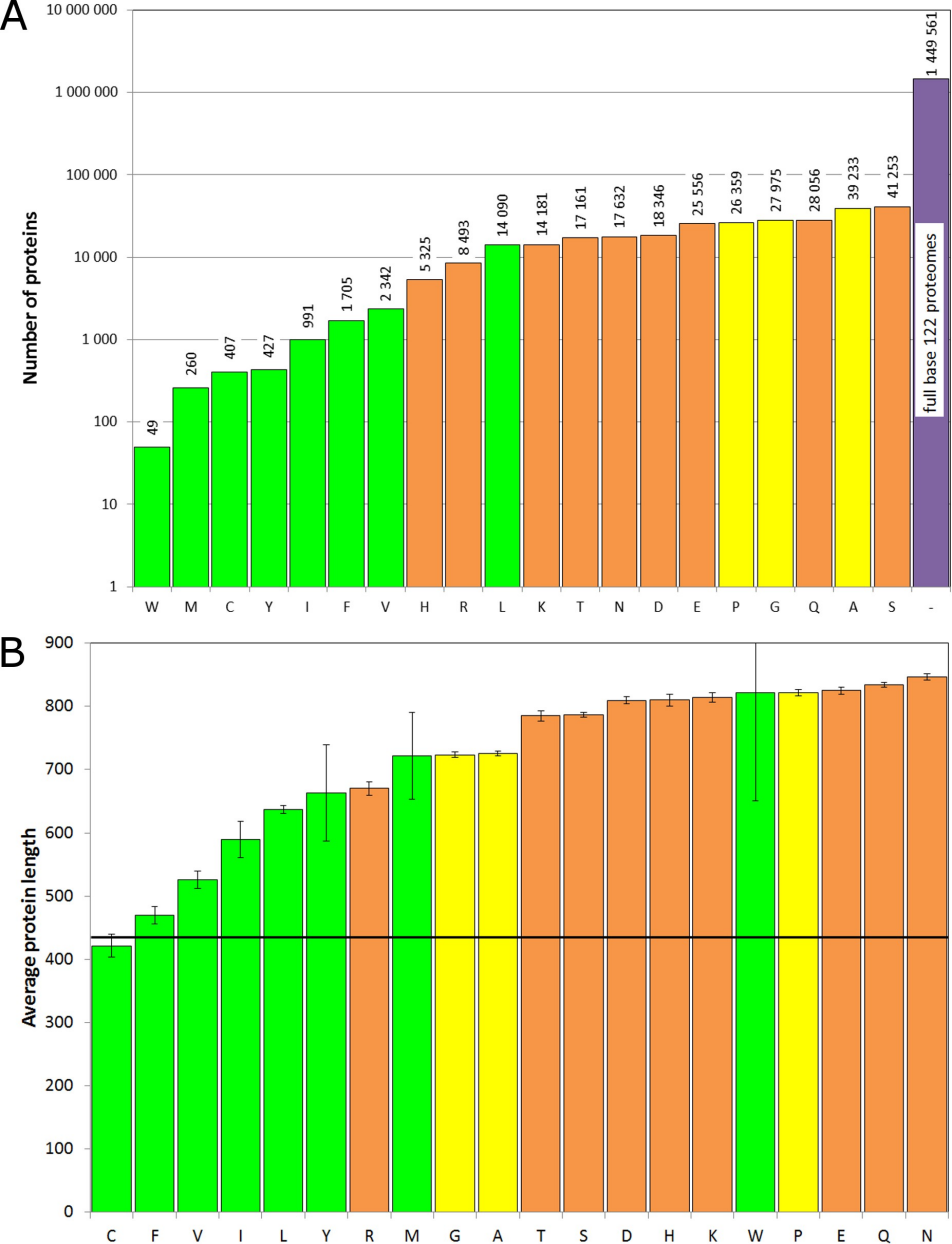
Properties of proteins with homo-repeats. **A**. Number of proteins with homo-repeats for 20 amino acids in 1 449 683 proteins from 122 proteomes. **B**. Averaged number of amino acid residues in proteins with homo-repeats for 20 amino acids.

In 215 481 proteins (15%) there are homo-repeats with the length of 5 residues and more. Our database includes 380 853 (*N*_*h*_) homo-repeats for all amino acids. The leader among these homo-repeats is serine. There are 41 253 serine homo-repeats, and only 49 tryptophan ones. The rest values are presented in Fig 1A. First, let us examine common features of proteins with homo-repeats.

As seen, the number of proteins with homo-repeats is less than the number of homo-repeats, because some homo-repeats occur in pairs. Green color corresponds to hydrophobic amino acids, orange to hydrophilic and charged ones, and yellow to small amino acids and proline. Hydrophobic homo-repeats occur rarer than the others with the exception of leucine.

Proteins with homo-repeats are on average longer than in the whole database. The average length of proteins in the database is 435 residues (shown by the bold line in Fig 1B), the average length of a protein with homo-repeats ranging from 421 for cysteine homo-repeats to 847 for asparagine homo-repeats. The differences between the average length proteins with homo-repeats and the average length of proteins in the whole database are significant for all with exception of C, F, W, Y, M. The statistical significance was estimated with the Z-score. The distribution of Z-scores can be approximated by a normal distribution. For isoleucine homo-repeat this difference is 5 standard deviations (s.d.), and the probability for this is less than $10^{-6}$; for V it is 7 s.d. and the probability is less than $10^{-10}$. For all the rest the difference is more than 20 s.d. and the probability of an accidental match is too small to count. It should be mentioned that the longer the protein the longer homo-repeat will be.

**Fig 2:**
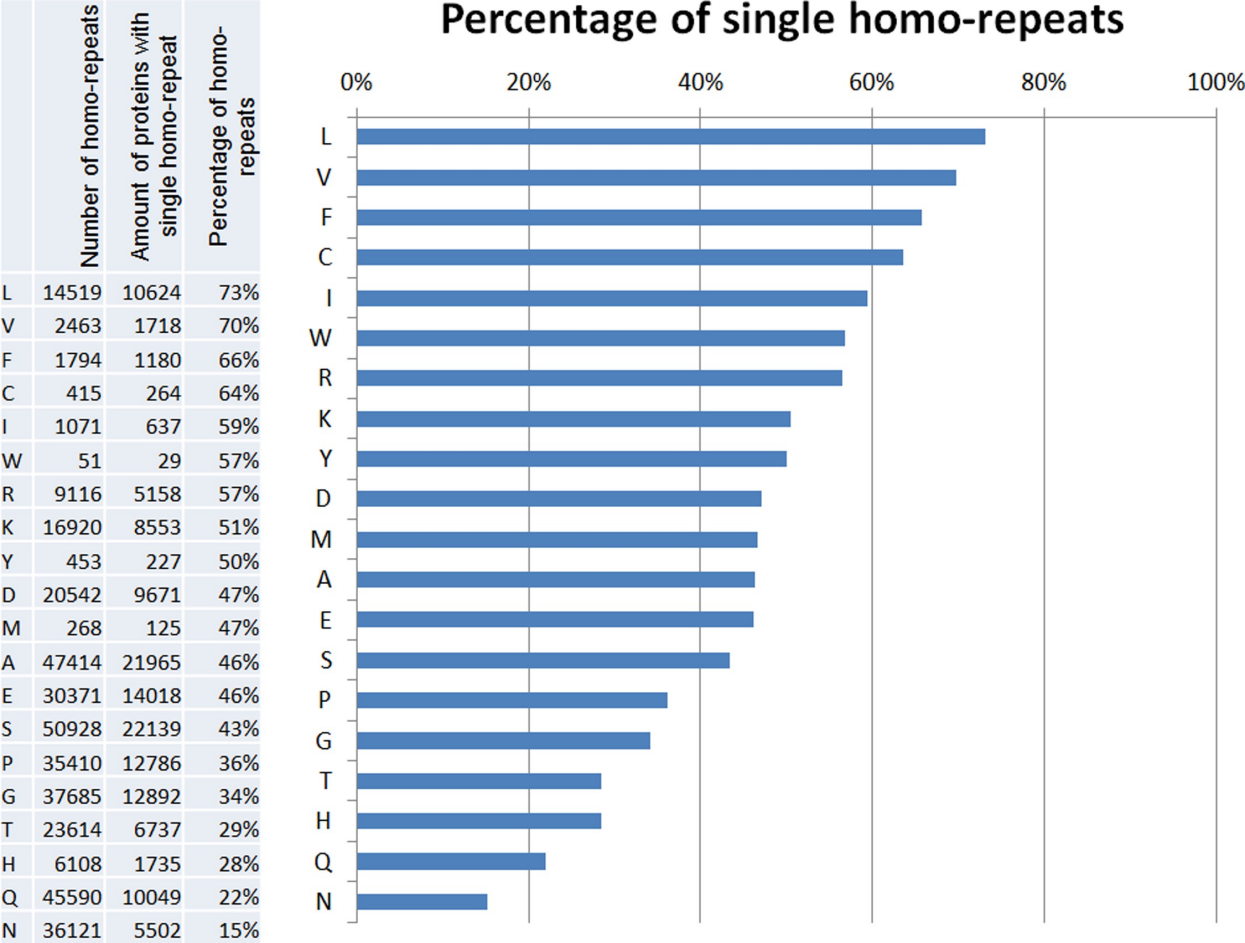
Fraction of single homo-repeats for 20 amino acids occurring in the proteins from 122 proteomes.

The percentage of single homo-repeats among all possible ones is presented in Fig 2. If the homo-repeats occur independently of each other in proteins, the proportion of single homo-repeats would be ${\left(1-\frac{1}{N_{p}}\right)}^{N_{h}}*100\approx 77$ for all amino acids. Meanwhile, even for leucine homo-repeats it is less (73%), although only slightly. But 15% of asparagine homo-repeats are not random. The number of proteins that have at least a couple of homo-repeats for two amino acids is shown in Table 1.

Different style is given according to the Z-values:

$$Z_{ij}=\frac{N_{ij}-N_{i}N_{j}/N_{p}}{{\left(N_{i}N_{j}/N_{p}\right)}^{1/2}}.$$ (1)

Here $N_{ij}$ is the number of proteins with homo-repeats for a pair of amino acids *i* and *j*. $N_{i}$ and $N_{j}$ are the numbers of homo-repeats for amino acids *i* and *j*, respectively. $N_{p}$ is the number of proteins in the database. Bold fontcorresponds to $Z_{ij}>50$, and italic font to $10\leq Z_{ij}\leq 50$. It is easy to note that the most striking result corresponds to the diagonal of the matrix, i.e., homo-repeats of the same amino acids are often found in pairs in the considered proteins. Moreover, the matrix is divided in two parts: the first one is the cluster of hydrophobic amino acids (CMFILVWY) and the second one includes small and hydrophilic amino acids (AGTSQN EDHRKP). The obtained result that hydrophobic amino acids prefer to occur in pair with hydrophobic ones, and polar, charged and small amino acids in pair with similar amino acids agrees with our previous result that the appearance of the first will decrease the fraction of the disordered residues, at the same time the occurrence of the second will increase the fraction of the disordered residues [7].

Large cluster with small, polar and charge amino acids again divided into 6 smaller clusters. A, G, T, S, Q, N prefer to appear in the same proteins. E and D prefer to appear together, H, R, and K prefer to be in pair with itself. P prefer to be with A, G, Q and P.

It should be noted that basic amino acid homo-repeats (R and K) are not very often combined with other homo-repeats, but are more common than one could randomly expect. The general result is that homo-repeats occur in pairs in the protein chain.

### Homo-repeats are important for prion-like domains of RNA-binding proteins

**Fig 3:**
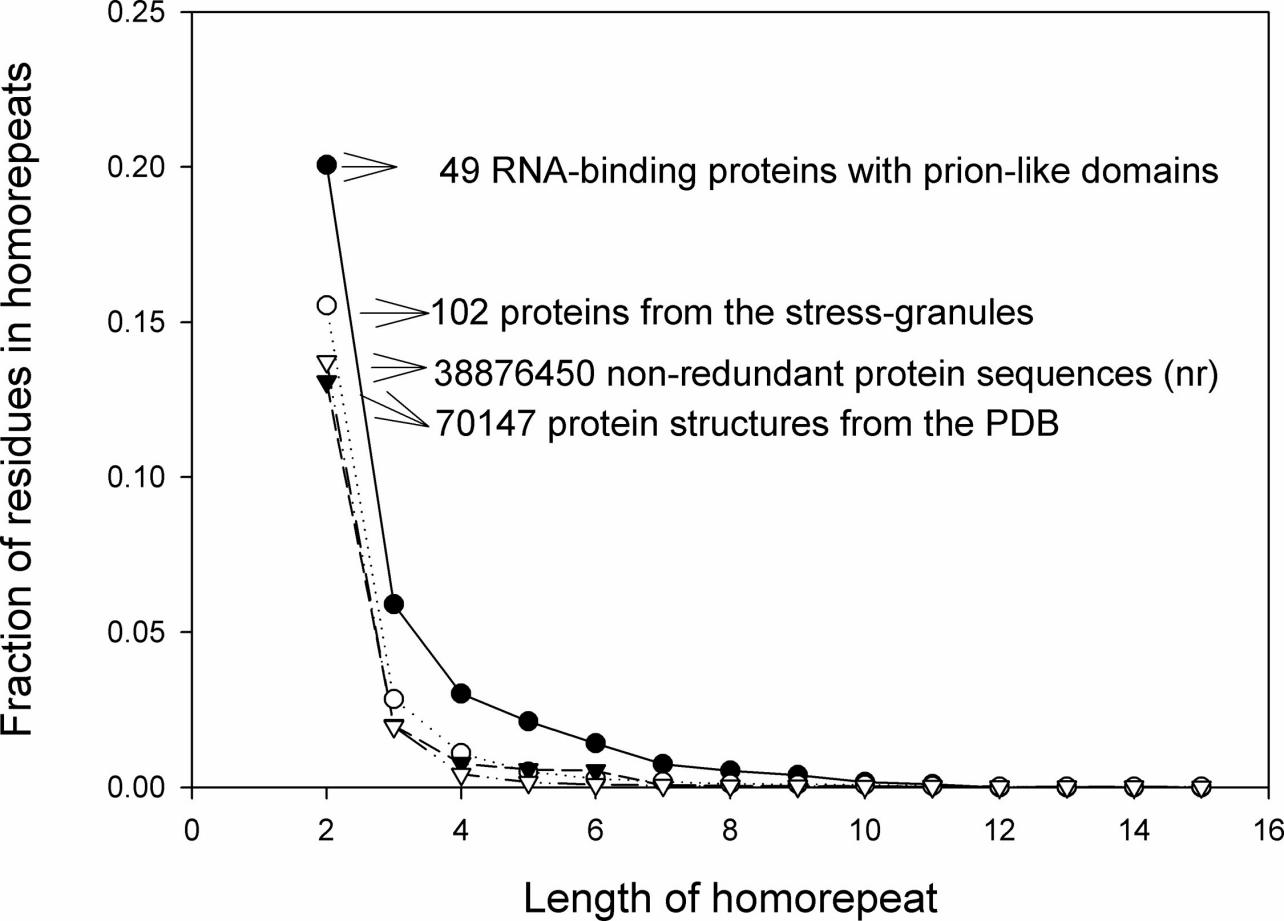
Occurrence of homo-repeats in the different set of proteins. Fraction of amino acid residues in homo-repeats versus the length of homo-repeats for 49 RNA-binding proteins with predicted prion-like domains (black circles), 102 proteins from stress granules (white circles), for 70 147 protein structures from the PDB (black triangles), and from the non-redundant 38 876 450 protein sequences (white triangles).

The formation of stress granules and all membrane less compartments (P-bodies, etc…) is considered a composition-driven molecular process. Many of the RNA-binding proteins that make up stress granules have prion-like domains. To verify that homo-repeats are important for some proteins, we considered two databases. One database consists of 49 RNA-binding proteins containing predicted prion-like domains published in [26]. These proteins enriched in some amino acids (see S1 Table). Prion-like domains are predominantly associated with enrichment of Q or N residues [27]. The other database is compiled from the Uniprot in which it is indicated that these proteins are included in the stress granules from the human proteome. In total 102 such proteins have been found. In order to compare these bases, we analyzed PDB (70 147 structures and non-redundant protein sequences (nr) 38 876 450). We estimated the fraction of amino acid residues included in the homo-repeats. We started from the length two, because it is the minimal length of any homo-repeat. It turned out that the fraction of amino acid residues in homo-repeats is larger for RNA-binding proteins with prion-like domains and for 102 proteins from the stress granules than for 70147 protein structures from the PDB, and from the non-redundant 38 876 450 protein sequences until 6 residue length for 49 RNA-binding proteins with prion-like domain and until 3 for 102 human proteins from the stress granules (Fig 3). It is important to underline that RNA-binding proteins with a prion-like domain involved in many protein functions and diseases are connected with misfolding of these proteins.

### Influence of homo-repeats on the aggregation properties of proteins

To examine whether homo-repeat enrichment can affect protein aggregation we explored the relationship between enrichment for each amino acid homo-repeat and aggregating properties of proteins. We describe the aggregating properties of proteins considering such the aggregation values as Spos, Sneg and Sall (see Material and methods) for each amino acid residue along the protein sequence using the FoldAmyloid program [28,29]. Comparison of the results for 30 proteins [30] using eight different methods demonstrated that our method is among the best ones (see Table 2).

**Table 2:** Averaged results of amyloid predictions (amyloidogenic regions) for 30 proteins by various algorithms.

Scoring type	PASTA2 [31]	Amyl Pred2 [32]	Tango [33]	Met Amyl [34]	Waltz [35]	Fold Amyloid [29]	Arch candy [36]	FISH-Amyloid [37]
Sensitivity	0.36	0.41	0.19	0.38	0.19	0.28	0.16	0.13
Specificity	0.91	0.86	0.95	0.86	0.94	0.92	0.92	0.95
False regions predicted as amyloidogenic	38	121	37	88	37	31	15	49
Number of correctly predicted regions / **total**	33/**46**	42/**46**	17/**46**	33/**46**	22/**46**	29/**46**	8/**46**	21/**46**

All methods were used under conditions of optimal specificity; FoldAmyloid was used with a sliding window of seven residues.

Also, it should be mentioned the review of Chiti who presented experimental data about the possibility of different methods of predictions of amyloidogenic regions *in vivo* [38]. He also demonstrated that our method is among the best methods. Recently, 14 different methods for the prediction of protein aggregation propensity have been considered [39].

**Fig 4:**
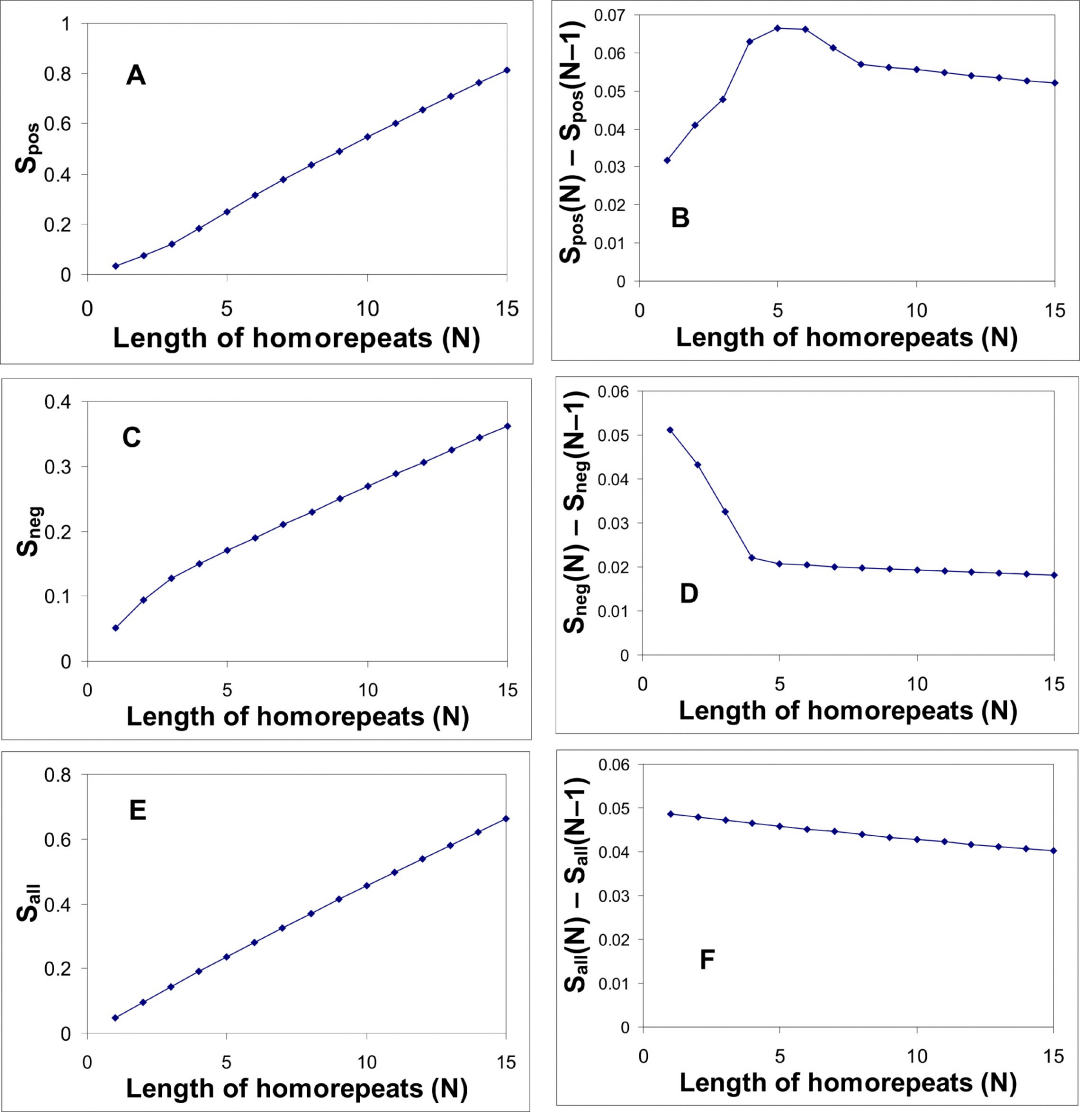
Effect of the single cysteine homo-repeat insertion of different length into the random proteome on Spos, Sneg, and Sall.

To observe the impact of homo-repeat in a pure form we performed an additional analysis to understand what properties of the protein chain will be changed after adding homo-repeats in the random sequences and the real proteins from 122 proteomes. To each protein in two bases (random proteome and 122 real proteomes) 20*15 homo-repeats have been added with the length from 1 to 15 residues. Homo-repeats are added in the middle of the chain. If the length of the protein represented an odd number of residues, then a homo-repeat was added between residues M and M+1 (2M+1 = N is the length of the given protein). The difference between Spos (N)—Spos(N-1) is shown in Fig 4. Sneg and Sall were treated by the same procedure (see Fig 4). Spos is the sum of significant positive peaks normalized by the length of the protein. When we add a homo-repeat the length of the protein increases. Therefore, Spos decreases when we add homo-repeat containing hydrophilic amino acids. And likewise the absolute value decreases Sneg when we add homo-repeat with hydrophobic amino acids.

To find the pure influence of a homo-repeat in protein we have added in all sequences, including 2 000 000 random sequences, artificial homo-repeat of different length from 1 and to 15 residues. The maximal effect which we observed for any homo-repeat corresponds to homo-repeat of 5–6 residues long. This result is consistent with the experimental observation that the minimal amyloidogenic fragment has also 5–6 residues. We present results only for cysteine because the results for other amino acids are similar (see S2 Table). For homo-repeats with hydrophilic amino acids the sign and graphs Sneg and Spos are reversed. Through this study, we can estimate the effect of the single homo-repeat on Spos, Sneg, and Sall. The dependences are the same for random and real 122 proteomes (S2 and S3 Tables).

In order to estimate the effect of homo-repeats themselves, we cut the longest homo-repeat for the given amino acid, and then recalculated the Spos, Sneg, and Sall for the protein chain without it. Finally, to assess the impact of all homo-repeats in the considered protein, we also cut out all homo-repeats and recalculated Spos, Sneg, and Sall again.

We can observe the influence of homo-repeats on the aggregation properties by looking from the other side: deleting the main homo-repeat in the first case and then deleting all homo-repeats from the protein.

After characterization of proteins with homo-repeats, we analyzed the aggregation properties of such proteins. For all proteins, we calculated Spos which reflects aggregation properties of proteins. The trivial effect is connected with the occurrence of hydrophobic home-repeats which will enhance the aggregation properties of protein by itself.

**Fig 5:**
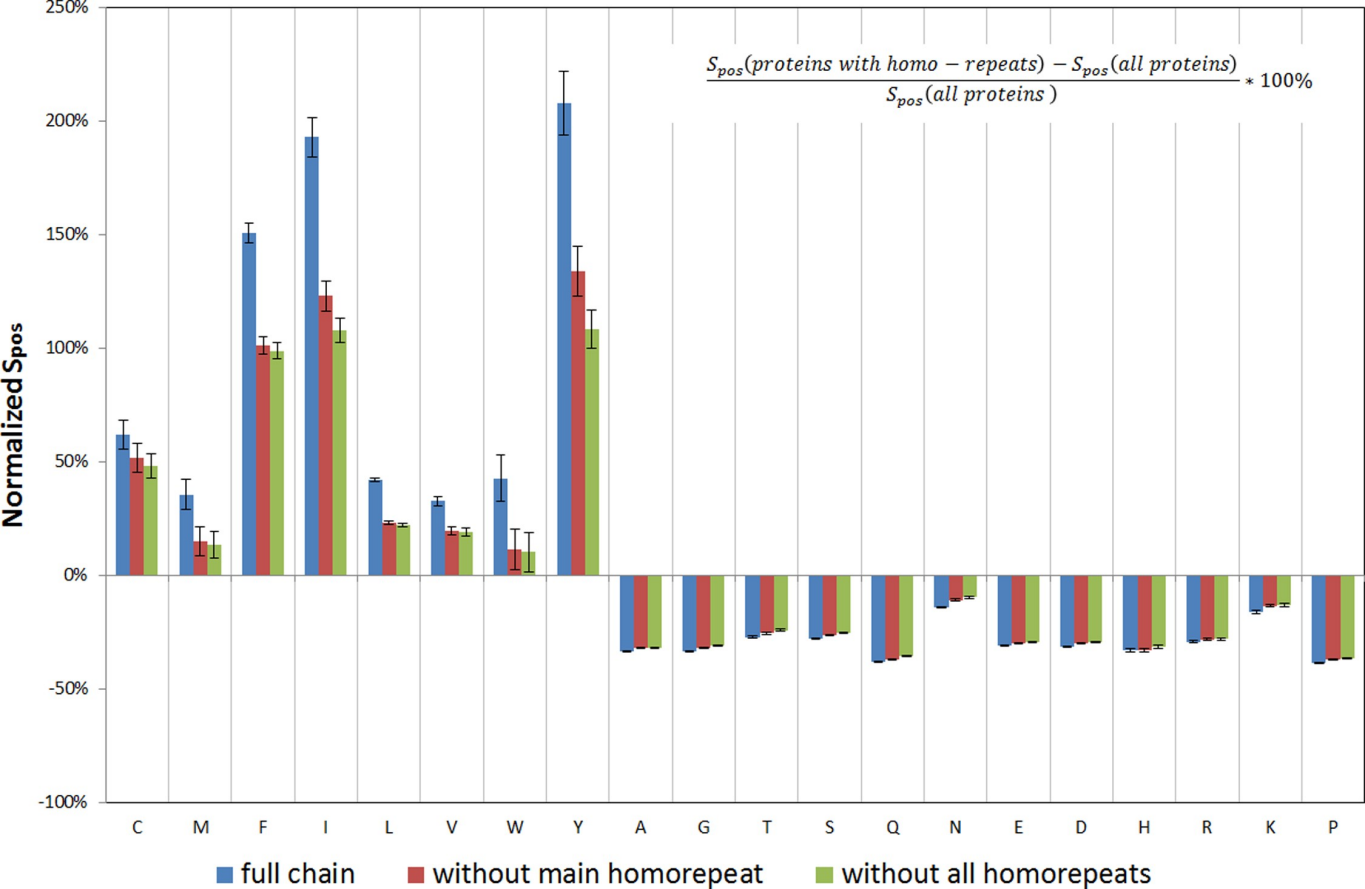
Comparison of normalized Spos scores for proteins with homo-repeats with the whole database. Blue bars correspond to normalized Spos scores for a full chain, red bars correspond to Spos scores for a chain without the main homo-repeat, and green bars correspond to Spos scores for a chain without all homo-repeats.

**Fig 6:**
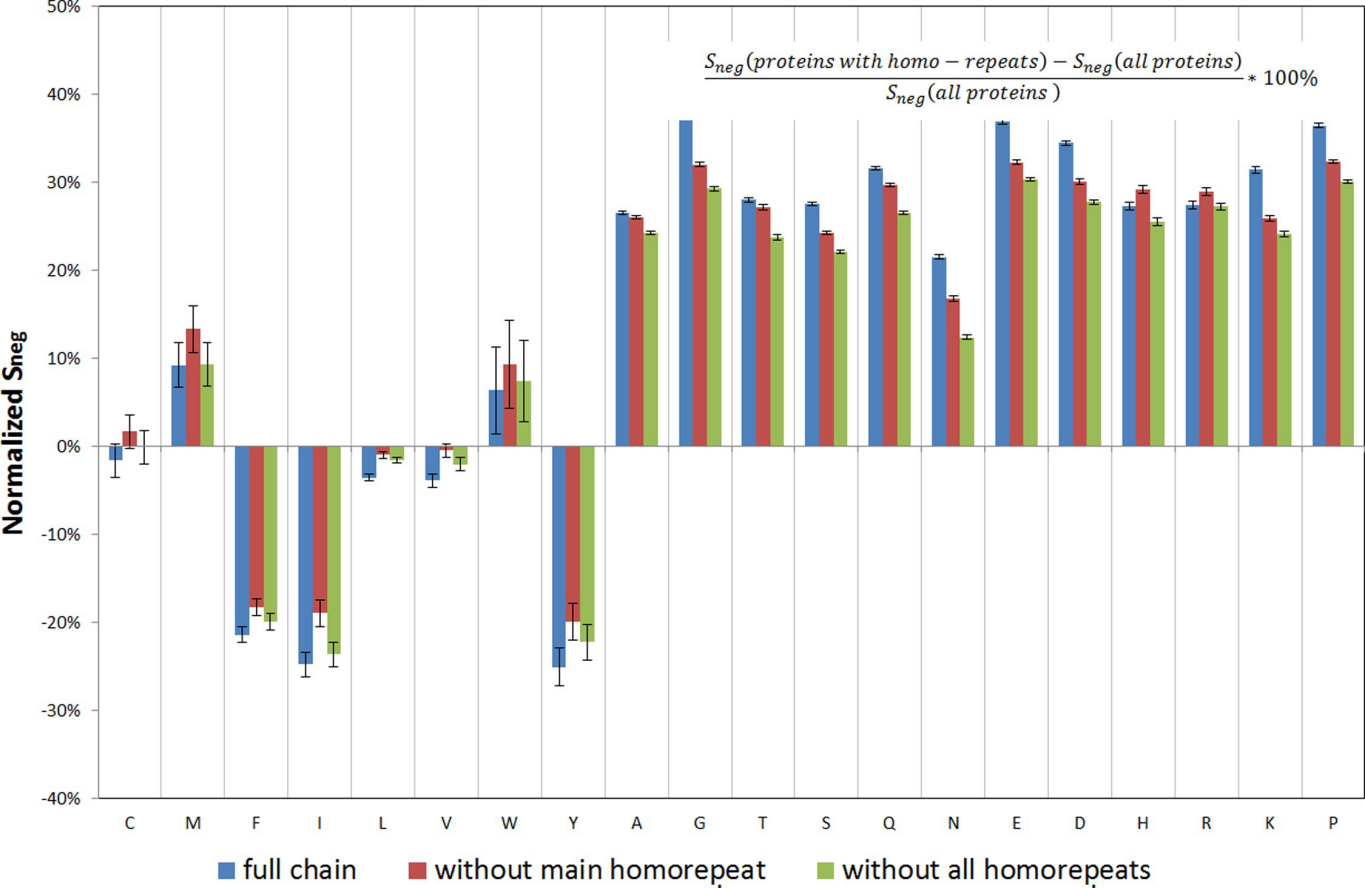
Comparison of normalized Sneg scores for proteins with homo-repeats and the whole database.

**Fig 7:**
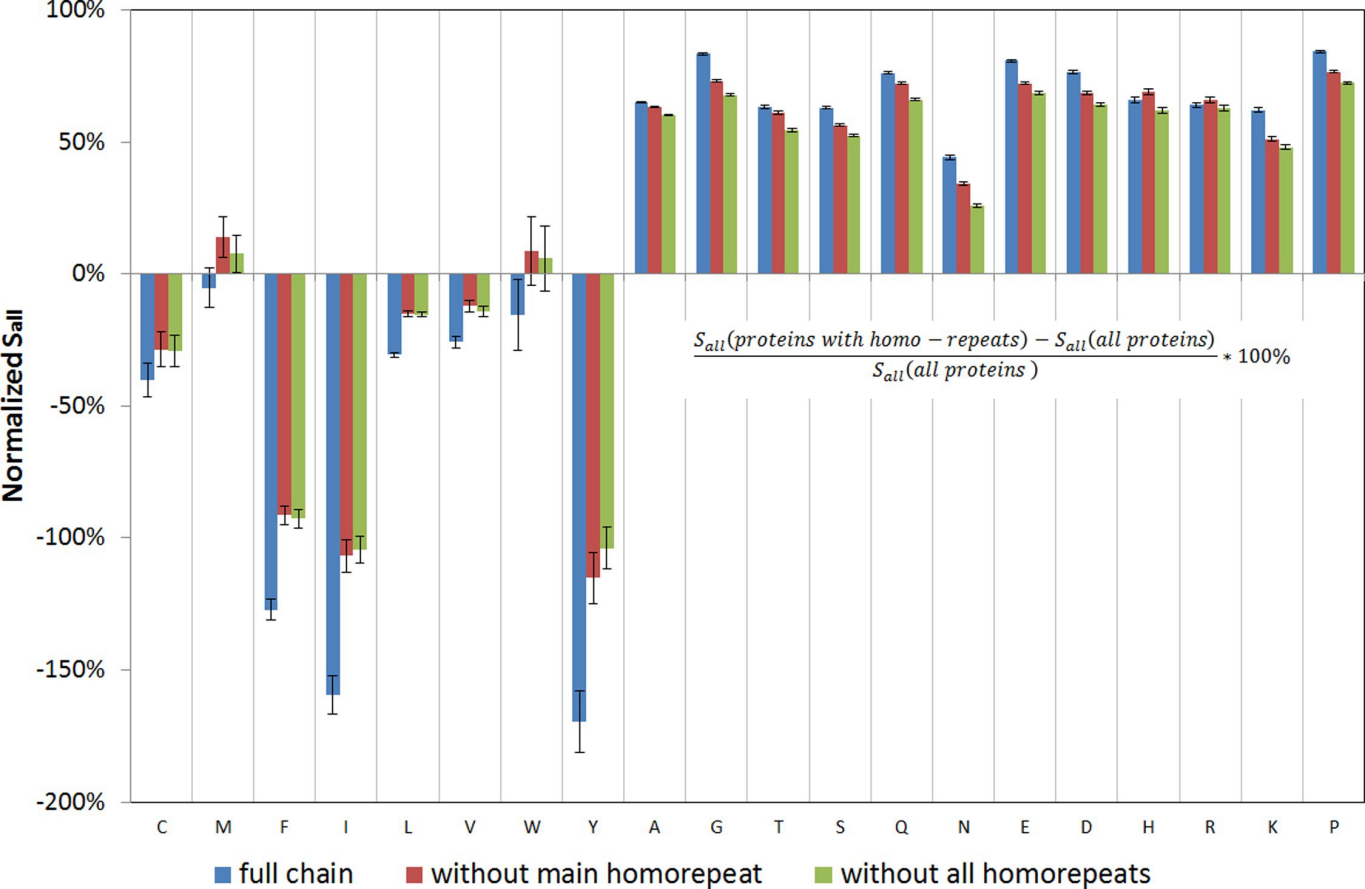
Comparison of normalized Sall scores for proteins with homo-repeats and the whole database.

The difference between Spos, Sneg, and Sall for proteins with homo-repeats and the entire database cannot be explained only by the occurrence of homo-repeats (Fig 5, data for Sneg, and Sall are presented in Figs 6 and 7). It is evident that for tryptophan and methionine, all the features are exhausted by the longest homo-repeat (Fig 5) (Spos decreases to zero after cutting off the main homo-repeat). But for all other amino acids, the difference between proteins with homo-repeats and the rest of the database is much larger than the impact of actual homo-repeats (Fig 5). Such a way we have demonstrated that homo-repeats enrichments influence on the protein aggregation properties.

In this paper, we have demonstrated the influence of homo-repeats with lengths larger than four amino acid residues on the aggregation properties of their host proteins considering 122 eukaryotic and bacterial proteomes. It turned out that proteins with homo-repeats are twice longer than the average length of proteins from 122 proteomes. We have shown that the aggregation properties of proteins with homo-repeats cannot be explained only by the appearance of the main (the longest) homo-repeat in the sequence. We have discovered that, as a rule, homo-repeats occur in pairs in the proteins, though hydrophobic and aromatic homo-repeats most frequently occur in pairs with similar ones, and homo-repeats constructed of polar, charged and small amino acids are prone to be in pair with similar homo-repeat. Considering different sets of proteins, we have demonstrated that the RNA-binding proteins with a prion-like domain have the maximal fraction of homo-repeats in comparison with those in the PDB and non-redundent dataset of sequences.

## Materials and methods

### FoldAmyloid program

**Fig 8:**
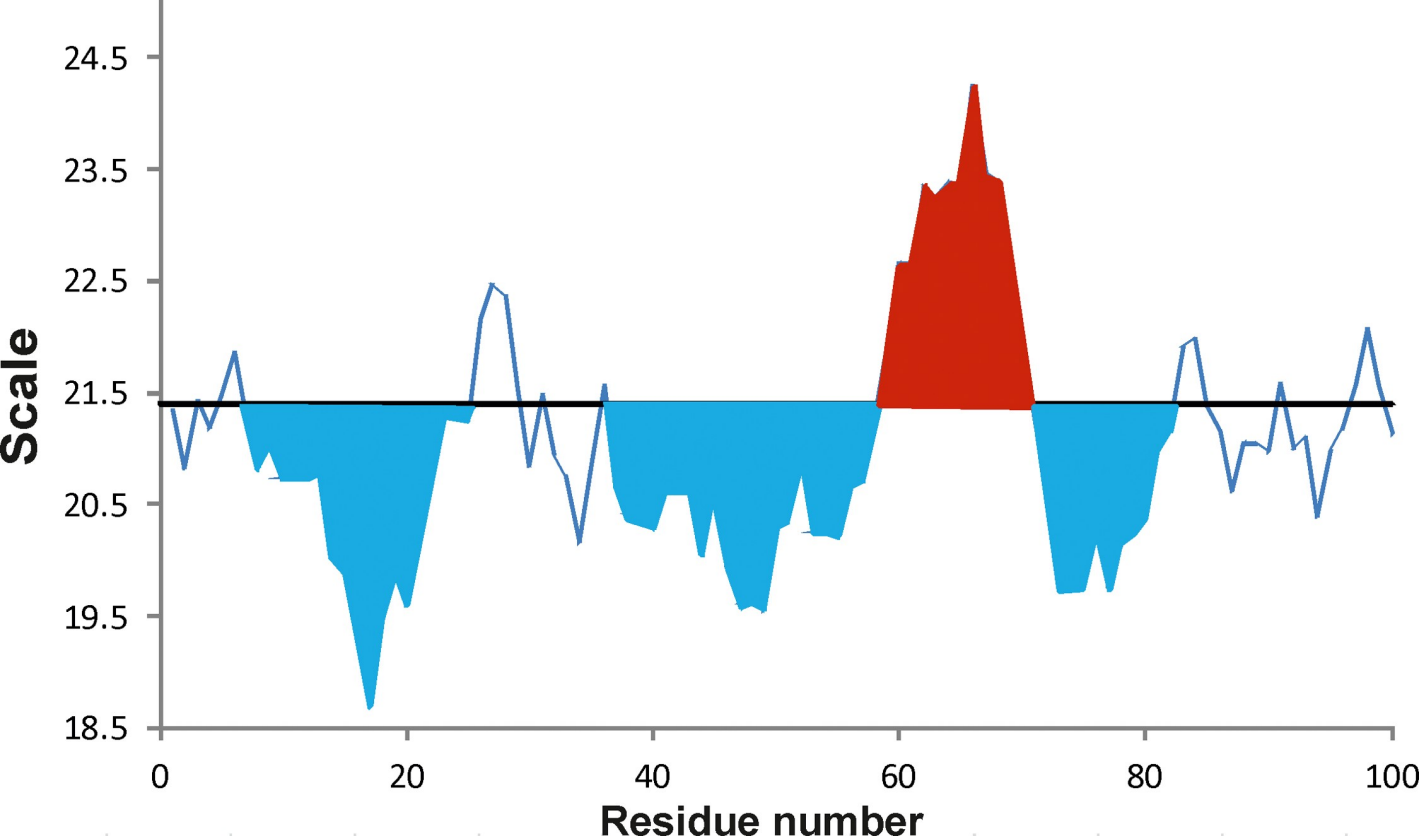
Schematic representation of amyloidogenic profile. The area under the peak that lies above the threshold of 21.4 is colored by red and below the threshold by blue.

The FoldAmyloid web server is available at http://bioinfo.protres.ru/fold-amyloid/. The program/server takes an amino acid sequence (in the FASTA format) as an input and calculates the profile of the requested type [in this case we used the scale of the expected number of contacts]. If five or more residues in the profile lie above the given cutoff (the default value is 21.4 for the packing density scale), we predict this region as amyloidogenic. Spos is the sum of areas of aggregation peaks, i.e. the area under the peak that lies above the threshold of 21.4, which is then normalized by the protein length (Fig 8). Sneg is the sum of areas of aggregation peaks that lies below the threshold of 21.4. Sall is the sum of aggregation values for each amino acid along the protein chain normalized by the protein length.

### Databases and programs

The HRaP database (http://bioinfo.protres.ru/hrap/) includes 1 449 683 proteins from 122 proteomes. For 215 481 proteins having homo-repeats the user can find the GO annotation. Also, we have considered the set of 49 RNA-binding proteins with predicted prion-like domains by using the prion score [39], 102 proteins from the stress granules, 38 876 450 non-redundant protein sequences and 70 147 protein structures from the PDB.

**Fig 9:**
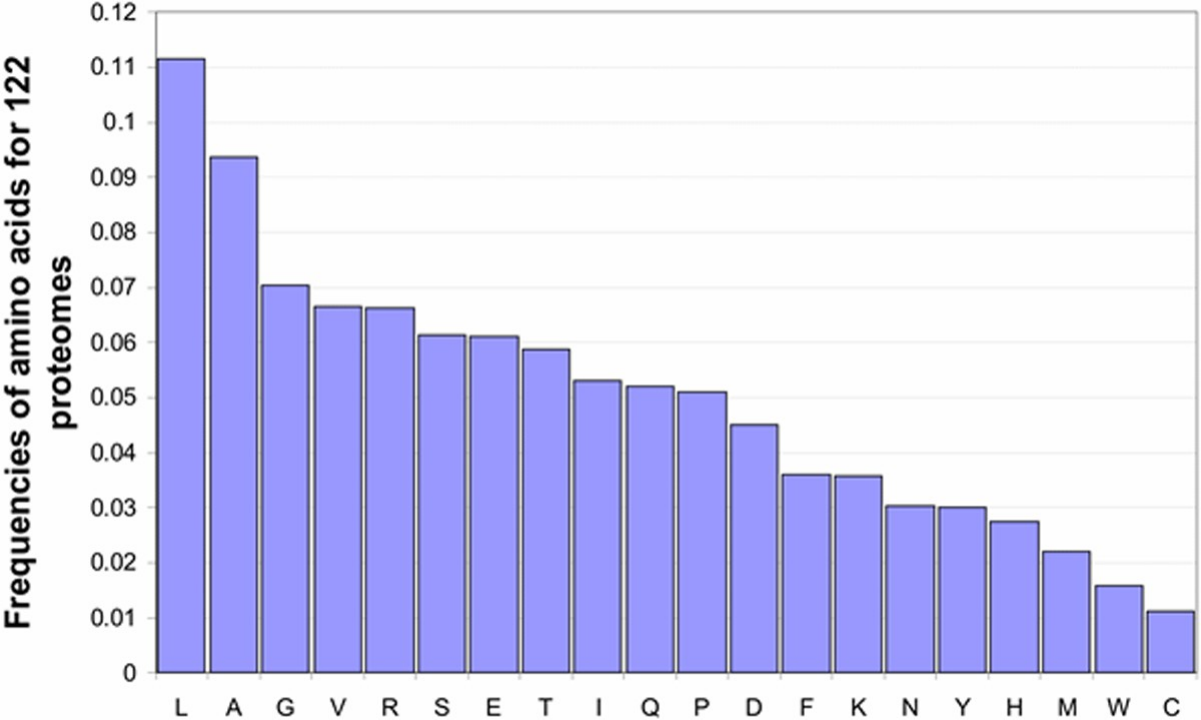
Frequencies of amino acids for 1449683 proteins from 122 proteomes.

The random proteome includes 2 000 000 sequences. The lengths of sequences vary from 50 to 550 amino acid residues. An amino acid was chosen randomly according to the frequencies of amino acids obtained from the real 122 proteomes (see Fig 9).

We used the database of 30 proteins and peptides to test the work of different programs that are not created by us [31]: prolactin, calcitonin, apolipoprotein A-I, casein, serum amyloid A1 protein, transthyretin, lactoferrin, semenogelin-1, Aβ42, gelsolin, tau, amylin, lung surfactant, α-synuclein, lysozyme, β2-microglobulin, medin, brain natriuretic peptide, apolipoprotein C-II, odontogenic ameloblast-associated protein, cystatin C, insulin chain A, insulin chain B, β-lactoglobulin, acylphosphatase-2, high mobility group protein B1, cold shock protein, kerato-epithelin, myoglobin, replication protein.

## Supporting information

S1 Table Amino acid composition values for 49 RNA-binding proteins with predicted prion-like domains. (XLSX)

S2 Table Effect of the single homo-repeat insertion of different length into the random proteome on Spos, Sneg, and Sall for 20 amino acids. (XLSX)

S3 Table Effect of the single homo-repeat insertion of different length into the proteins from 122 proteomes on Spos, Sneg, and Sall for 20 amino acids. (XLSX)

## References

[1] Siwach P, Ganesh S. Tandem repeats in human disorders: mechanisms and evolution. Front Biosci J Virtual Libr. 2008;13: 4467–4484. 10.2741/3017 18508523

[2] Lobanov MY, Klus P, Sokolovsky IV, Tartaglia GG, Galzitskaya OV. Non-random distribution of homo-repeats: links with biological functions and human diseases. Sci Rep. 2016;6: 26941 10.1038/srep26941 27256590 PMC4891720

[3] Jorda J, Xue B, Uversky VN, Kajava AV. Protein tandem repeats—the more perfect, the less structured. FEBS J. 2010;277: 2673–2682. 10.1111/j.1742-464X.2010.07684.x 20553501 PMC2928880

[4] Lobanov MY, Furletova EI, Bogatyreva NS, Roytberg MA, Galzitskaya OV. Library of disordered patterns in 3D protein structures. PLoS Comput Biol. 2010;6: e1000958 10.1371/journal.pcbi.1000958 20976197 PMC2954861

[5] Lobanov MY, Galzitskaya OV. Occurrence of disordered patterns and homorepeats in eukaryotic and bacterial proteomes. Mol Biosyst. 2012;8: 327–337. 10.1039/c1mb05318c 22009164

[6] Lobanov MY, Galzitskaya OV. Disordered patterns in clustered Protein Data Bank and in eukaryotic and bacterial proteomes. PloS One. 2011;6: e27142 10.1371/journal.pone.0027142 22073276 PMC3208572

[7] Lobanov MY, Galzitskaya OV. How Common Is Disorder? Occurrence of Disordered Residues in Four Domains of Life. Int J Mol Sci. 2015;16: 19490–19507. 10.3390/ijms160819490 26295225 PMC4581309

[8] Darling A, Uversky V. Intrinsic Disorder in Proteins with Pathogenic Repeat Expansions. Molecules. 2017;22: 2027 10.3390/molecules22122027 29186753 PMC6149999

[9] Fan X, Dion P, Laganiere J, Brais B, Rouleau GA. Oligomerization of polyalanine expanded PABPN1 facilitates nuclear protein aggregation that is associated with cell death. Hum Mol Genet. 2001;10: 2341–2351. 11689481 10.1093/hmg/10.21.2341

[10] Mularoni L, Ledda A, Toll-Riera M, Albà MM. Natural selection drives the accumulation of amino acid tandem repeats in human proteins. Genome Res. 2010;20: 745–754. 10.1101/gr.101261.109 20335526 PMC2877571

[11] Robertson AL, Bate MA, Androulakis SG, Bottomley SP, Buckle AM. PolyQ: a database describing the sequence and domain context of polyglutamine repeats in proteins. Nucleic Acids Res. 2011;39: D272–276. 10.1093/nar/gkq1100 21059684 PMC3013692

[12] Cascarina SM, Ross ED. Proteome-scale relationships between local amino acid composition and protein fates and functions. PLoS Comput Biol. 2018;14: e1006256 10.1371/journal.pcbi.1006256 30248088 PMC6171957

[13] Monsellier E, Ramazzotti M, Taddei N, Chiti F. Aggregation propensity of the human proteome. PLoS Comput Biol. 2008;4: e1000199 10.1371/journal.pcbi.1000199 18927604 PMC2557143

[14] Tartaglia GG, Caflisch A. Computational analysis of the S. cerevisiae proteome reveals the function and cellular localization of the least and most amyloidogenic proteins. Proteins. 2007;68: 273–278. 10.1002/prot.21427 17407164

[15] de Groot NS, Ventura S. Protein aggregation profile of the bacterial cytosol. PloS One. 2010;5: e9383 10.1371/journal.pone.0009383 20195530 PMC2828471

[16] Prusiner SB, editor. Prion biology and diseases. 2nd ed Cold Spring Harbor, N.Y: Cold Spring Harbor Laboratory Press; 2004.

[17] Flechsig E, Shmerling D, Hegyi I, Raeber AJ, Fischer M, Cozzio A, et al Prion protein devoid of the octapeptide repeat region restores susceptibility to scrapie in PrP knockout mice. Neuron. 2000;27: 399–408. 10985358 10.1016/s0896-6273(00)00046-5

[18] Liu JJ, Lindquist S. Oligopeptide-repeat expansions modulate “protein-only” inheritance in yeast. Nature. 1999;400: 573–576. 10.1038/23048 10448860

[19] Krishnan R, Lindquist SL. Structural insights into a yeast prion illuminate nucleation and strain diversity. Nature. 2005;435: 765–772. 10.1038/nature03679 15944694 PMC1405905

[20] Galzitskaya OV. Repeats are one of the main characteristics of RNA-binding proteins with prion-like domains. Mol Biosyst. 2015;11: 2210–2218. 10.1039/c5mb00273g 26022110

[21] Wright CF, Teichmann SA, Clarke J, Dobson CM. The importance of sequence diversity in the aggregation and evolution of proteins. Nature. 2005;438: 878–881. 10.1038/nature04195 16341018

[22] López de la Paz M, Serrano L. Sequence determinants of amyloid fibril formation. Proc Natl Acad Sci U S A. 2004;101: 87–92. 10.1073/pnas.2634884100 14691246 PMC314143

[23] Thompson MJ, Sievers SA, Karanicolas J, Ivanova MI, Baker D, Eisenberg D. The 3D profile method for identifying fibril-forming segments of proteins. Proc Natl Acad Sci U S A. 2006;103: 4074–4078. 10.1073/pnas.0511295103 16537487 PMC1449648

[24] de Groot NS, Parella T, Aviles FX, Vendrell J, Ventura S. Ile-phe dipeptide self-assembly: clues to amyloid formation. Biophys J. 2007;92: 1732–1741. 10.1529/biophysj.106.096677 17172307 PMC1796831

[25] Lobanov MY, Sokolovskiy IV, Galzitskaya OV. HRaP: database of occurrence of HomoRepeats and patterns in proteomes. Nucleic Acids Res. 2014;42: D273–278. 10.1093/nar/gkt927 24150944 PMC3965023

[26] Li YR, King OD, Shorter J, Gitler AD. Stress granules as crucibles of ALS pathogenesis. J Cell Biol. 2013;201: 361–372. 10.1083/jcb.201302044 23629963 PMC3639398

[27] Alberti S, Halfmann R, King O, Kapila A, Lindquist S. A Systematic Survey Identifies Prions and Illuminates Sequence Features of Prionogenic Proteins. Cell. 2009;137: 146–158. 10.1016/j.cell.2009.02.044 19345193 PMC2683788

[28] Galzitskaya OV, Garbuzynskiy SO, Lobanov MY. Prediction of amyloidogenic and disordered regions in protein chains. PLoS Comput Biol. 2006;2: e177 10.1371/journal.pcbi.0020177 17196033 PMC1761655

[29] Garbuzynskiy SO, Lobanov MY, Galzitskaya OV. FoldAmyloid: a method of prediction of amyloidogenic regions from protein sequence. Bioinforma Oxf Engl. 2010;26: 326–332. 10.1093/bioinformatics/btp691 20019059

[30] Dovidchenko NV, Galzitskaya OV. Computational Approaches to Identification of Aggregation Sites and the Mechanism of Amyloid Growth. Adv Exp Med Biol. 2015;855: 213–239. 10.1007/978-3-319-17344-3_9 26149932

[31] Walsh I, Seno F, Tosatto SCE, Trovato A. PASTA 2.0: an improved server for protein aggregation prediction. Nucleic Acids Res. 2014;42: W301–307. 10.1093/nar/gku399 24848016 PMC4086119

[32] Tsolis AC, Papandreou NC, Iconomidou VA, Hamodrakas SJ. A consensus method for the prediction of “aggregation-prone” peptides in globular proteins. PloS One. 2013;8: e54175 10.1371/journal.pone.0054175 23326595 PMC3542318

[33] Fernandez-Escamilla A-M, Rousseau F, Schymkowitz J, Serrano L. Prediction of sequence-dependent and mutational effects on the aggregation of peptides and proteins. Nat Biotechnol. 2004;22: 1302–1306. 10.1038/nbt1012 15361882

[34] Emily M, Talvas A, Delamarche C. MetAmyl: a METa-predictor for AMYLoid proteins. PloS One. 2013;8: e79722 10.1371/journal.pone.0079722 24260292 PMC3834037

[35] Maurer-Stroh S, Debulpaep M, Kuemmerer N, Lopez de la Paz M, Martins IC, Reumers J, et al Exploring the sequence determinants of amyloid structure using position-specific scoring matrices. Nat Methods. 2010;7: 237–242. 10.1038/nmeth.1432 20154676

[36] Ahmed AB, Znassi N, Château M-T, Kajava AV. A structure-based approach to predict predisposition to amyloidosis. Alzheimers Dement J Alzheimers Assoc. 2015;11: 681–690. 10.1016/j.jalz.2014.06.007 25150734

[37] Gasior P, Kotulska M. FISH Amyloid–a new method for finding amyloidogenic segments in proteins based on site specific co-occurence of aminoacids. BMC Bioinformatics. 2014;15: 54 10.1186/1471-2105-15-54 24564523 PMC3941796

[38] Belli M, Ramazzotti M, Chiti F. Prediction of amyloid aggregation in vivo. EMBO Rep. 2011;12: 657–663. 10.1038/embor.2011.116 21681200 PMC3128957

[39] Pallarès I, Ventura S. Advances in the prediction of protein aggregation propensity. Curr Med Chem. 2017; 10.2174/0929867324666170705121754 28685682

